# The Closer, The Better? Processing Relations Between Picture Elements in Historical Paintings

**DOI:** 10.16910/jemr.13.2.11

**Published:** 2020-12-01

**Authors:** Manuela Glaser, Manuel Knoos, Stephan Schwan

**Affiliations:** Leibniz-Institut für Wissensmedien, Tübingen, Germany

**Keywords:** art perception, eye tracking, region of interest, attention, memory, historical paintings, spatial contiguity, text coherence, audio text

## Abstract

The present eye-tracking study investigated how audio explanations influence perception and the cognitive processing of historical paintings. Spatially close and distant pairs of picture elements and their semantic relations were named in an audio text either immediately after each other or with descriptions of other elements in between. It was assumed that the number of backward fixation counts on the first of the two mentioned related picture elements should be higher if they are spatially close rather than spatially distant. There should also be more backward fixation counts if the elements are named temporally close rather than temporally distant. Similar predictions were made for the retention of these picture elements and their relations. A 2x2x2 within-subject design (n=36) with spatial distance (close vs. distant), temporal distance (close vs. distant) and painting (Leutze vs. West) revealed more background fixation counts for spatially close compared to spatially distant elements but just for the Leutze painting. Accordingly, the relations between the spatially close pairs were retained better than between the spatially distant pairs in the Leutze painting but vice versa for the West painting. The results are discussed with regard to the spatial contiguity principle of multimedia learning and research on text coherence.

## Introduction

Paintings of mythological, religious, historical, or contemporary events typically depict complex scenes of persons, objects, and settings which stand in a more or less close relation to each other. In Benjamin West’s painting "The Death of General Wolfe" from 1770, for example, the following picture elements are thematically closely related to each other: The painting depicts Henry Browne, a lieutenant of the grenadier regiment holding the British flag, and a hat lying far from him on the ground. This kind of hat was typically worn by grenadiers like him. Further, the British flag depicted in the painting and a praying soldier next to it both symbolize a higher purpose and patriotism as justifications for the depicted battle.

West’s painting exemplifies two important points: Firstly, picture elements which are conceptually related are not necessarily placed close to each other but may instead be located at distant regions in a painting. Secondly, the implied conceptual relations between picture elements are often not readily apparent to lay viewers and may not guide their viewing behaviour in a free viewing situation. Therefore, museums often provide audio guides with verbal explanations that explicitly name picture elements and their conceptual relations in order to help visitors take a closer look at the artworks. Accordingly, when asked about the advantages of The National Gallery’s audio guide, visitors "were particularly positive about content designed to help them look at details they may otherwise have missed or take meaning from details that might otherwise have passed them by" (1).

While previous research has shown that accompanying verbal explanations help laypersons take notice of particular details of a painting (2, 3), the present study extends this line of research by investigating the role of concurrent verbal explanations on becoming aware of conceptual relations between picture elements in paintings. Based on theories of multimedia learning, we assume that the notion of "becoming aware" should not only be manifest in viewing behaviour but also in subsequent memory for pictorial details. In addition, the effect of naming two related picture elements in a concurrent verbal explanation on viewing behaviour and memory should be influenced both by the spatial distance of the respective elements in the painting and by the temporal distance between the naming of each of the elements in the verbal explanation.

According to the spatial contiguity principle of multimedia learning ( 4), written text and pictorial information should be mentally integrated more easily when presented spatially close since otherwise effortful visual search and memory processes are needed to perceptually and cognitively connect this information. This should also be the case for two picture elements. Therefore, conceptually related picture elements that are depicted spatially close to each other should be perceptually and cognitively better linked than picture elements that are spatially distant.

Many paintings of historical events are populated with a great number of persons and objects and allow for a large number of conceptual linkages between them. Accordingly, audio guide texts face the task of selecting some of these linkages and bringing them into a coherent sequence. Due to the spatial and nonlinear character of paintings, verbal explanations may nevertheless verbally link a picture element to another one that has already been mentioned several sentences before. In other words, audio texts accompanying a painting can name and explain picture elements and their conceptual relations either in immediate succession (that is, temporally close) or at different points in the narration (that is, temporally distant to each other). Research on text coherence shows that information is processed more rapidly and is therefore faster understood if related information instead of unrelated information precedes it (5). Hence, two pieces of related verbal information should be better cognitively integrated if they are presented in temporally close succession instead of a temporally distant presentation with several unrelated pieces of verbal information in between. Furthermore, since research using the blank screen paradigm indicates a close connection between the perceived picture scene, its verbal description, and its visual mental representation (6, 7), cognitive linkage of textual information also visible in the painting may be associated with corresponding perceptual linkages. Hence, providing explicit verbal descriptions should not only be helpful with regard to cognitive but also with regard to perceptual linkage of related picture elements if the related picture elements are named temporally close rather than distant to each other. The present study, therefore, examines the influence of temporally close versus temporally distant verbal explanations of conceptually related picture elements on perceptual and cognitive linkage of two related picture elements.

In the following, we will outline research on free viewing behaviour according to the information processing stage model of aesthetic processing by Leder, Belke, Oeberst, and Augustin (8) and the integrated model of text and picture comprehension by Schnotz (9). We will also review research especially on the spatial contiguity effect (4), giving insight into the processing of spatially close and distant picture elements. In addition, we will review theoretical and empirical literature on text comprehension, including the construction-integration model of text comprehension by Kintsch (10). This allows us to describe the processing of text elements that are close versus distant to each other. Based on these accounts, we will formulate hypotheses on the influence of spatial contiguity and text coherence on visual processing and memory of related picture elements and their relations.

### Processes of picture viewing

Free viewing of artworks is described in the information processing stage model of aesthetic processing by Leder et al. (8). On the cognitive level, they differentiate between an initial automatic processing and a subsequent deliberate processing. During automatic processing, the painting is perceptually analyzed with regard to aspects of the composition such as location, order and grouping of picture elements, as well as contrasts and symmetry. Next, these features are implicitly compared with previous experiences and, based on that, appear more or less familiar and prototypical to the viewer. During deliberate processing, the picture is explicitly classified with regard to style and content based on prior knowledge and interests. The picture is then interpreted in an art-specific and also in a self-related way, this cognitive mastering being constantly evaluated.

In multimedia research, the integrated model of text and picture comprehension by Schnotz (9) postulates similar processes taking place during picture viewing but with a focus on the interplay of attention, processing in working memory, and long-term storage, as well as on visualizations of scientific content, which includes realistic depictions as well as graphs and maps. According to this model, pictorial information enters the cognitive system via the visual channel and is analyzed with regard to its visual features, resulting in visuospatial patterns held in working memory, similar to the stage of automatic processing in the model by Leder et al. (8). The next step in the model by Schnotz (9) is a semantic deep structure processing that results in the construction and elaboration of a mental model of the picture’s content. The mental model serves as a core structure into which both corresponding verbal information, for example from audio texts, and lexical, perceptual, and conceptual knowledge from long-term memory is integrated. This semantic deep structure processing is similar to the deliberate explicit classification and interpretation processes in the model by Leder et al. (8). Compared to Leder et al. (8), the model by Schnotz (9) describes the cognitive structures involved in picture processing in more detail, but Leder et al. (8), on the other hand, goes beyond the mere generation of a picture-based mental model by addressing also meta-cognitive evaluation processes related to aesthetic judgements and aesthetic emotions as well as affective and contextual aspects of art perception.

According to multimedia theories, human memory is strictly resource limited and operates in parallel via at least two different channels, a verbal and a visuospatial one. Therefore, ease of processing paintings together with accompanying verbal descriptions strongly depends on the characteristics of the pictorial and verbal materials and their interplay. In the following, we will discuss two of these characteristics in more detail, namely, the effects of the spatial distance between related picture elements and the effects of the temporal distance of naming related picture elements in a concurrent verbal description on viewing behaviour and memory.

### Spatial distance between related picture elements

Based on theories about cognitive structures and their limited processing capacities in different processing channels such as the one by Schnotz (9), the spatial contiguity principle of multimedia learning ( 4) postulates that in order to reduce extraneous load (i.e. cognitive costs caused by the arrangement of the learning material), related elements in a visual presentation that are spatially close to each other should be better integrated in memory than elements which are spatially distant to each other. This spatial contiguity effect is well documented in previous research (for a meta-analysis see 11) but with a focus on combinations of written texts and related pictorial information in scientific learning material. Chandler and Sweller (12), for example, compared the written and practical training results of learners presented with learning material that contained illustrations with integrated (that is, spatially close) versus non-integrated (that is, spatially distant) textual information in an industrial training setting. They could show that test performance was better with spatially close than spatially distant combinations of textual and picture elements.

Furthermore, research on the spatial contiguity failure indicates that the influence of spatial contiguity on perceptual and cognitive linkage is independent of the semantic relation of the picture elements. Beege, Wirzberger, Nebel, Schneider, Schmidt, and Rey (13) varied the spatial distance between a pictorial presentation and related text labels (high vs. medium vs. low) and could show that retention and transfer performance was best with medium distance. Beege et al. ([Bibr b13]) argue that presenting labels too close to the related picture content leads also to a higher proximity between unrelated text and picture elements, hindering cognitive integration processes for related information. Hence, integration processes between two visual elements seem to be more probable the lower their spatial distance is, independent of whether they are semantically related or not.

Johnson and Mayer (14) examined the influence of spatial contiguity on visual attention in an eye tracking study. The eye movement behaviour of learners presented with integrated learning material about car brakes in which short textual descriptions were placed near to their corresponding areas in a diagram was compared to the eye movements of learners presented with separated learning material in which the texts were presented as a paragraph below the diagram. They could show that there were more transitional saccades between corresponding textual and picture elements in the spatially close condition than in the separated condition, indicating a better integration of spatially close elements on a perceptual level.

Similar effects of spatial distance have been found for picture-picture-comparisons by Bauhoff, Huff, and Schwan (15), who asked their participants to compare two depictions of pendulum clocks in order to detect differences between these depictions, varying their spatial distance. They could show a trade-off between eye movements and working memory use as strategies to do the task. While there was no significant effect of distance on the proportion of correct scores, with increasing distance participants showed a fewer number of gaze-shifts between the two depictions. This indicated a stronger perceptual linkage between spatially close compared to spatially distant picture elements.

This finding is in line with the results from artwork perception which demonstrate that perceptually establishing relations between distant picture elements is bound to viewers with sufficient art expertise. Since free viewing is not only based on bottom-up determinants included in the surface structure of the painting or the arrangement of the presentation but also on top-down aspects such as previous experiences, prior knowledge, and interests of the viewers, free picture viewing reveals large interindividual differences in the visual scan paths (16). Empirical studies about experts and laypersons cognitively processing paintings have shown that the influence of bottom-up determinants, such as the saliency of the picture elements, is reduced for experts compared to laypersons (17) and that the eye movement paths of experts are characterized by higher saccade lengths than the eye movement paths of laypersons (18, 19, 20). Such expertise counters tendencies to spontaneously focus on elements that are located in the middle of the painting, are perceptually salient, or possess a high relevance (like faces).

This indicates that when looking at a painting, experts compared to lay viewers visually and cognitively associate spatially distant picture elements in order to arrive at an adequate understanding of the picture. For example, it is important for the understanding of Renaissance portraits to consider the meaning of the symbolic objects usually depicted in the periphery of the paintings (21) and relate them to the centrally depicted person. One reason for this difference in eye movement behaviour may be that laypersons lack the prior knowledge of the meaning of such symbols or other types of relations between picture elements.

### Temporal distance between naming related picture elements

In order to help lay viewers to perceptually and cognitively link distant picture elements, an accompanying verbal explanation (e.g., an audio or personal guide in a museum) may be used in which the picture elements are named, described, interpreted, and related to each other. If such a text is appropriately formulated, it will guide the viewers' attention through the painting, helping them to notice particular elements and to establish semantic and formal relations (1, 3).

In order to fulfill this goal, texts should be structured in a way that enables the fluent generation of a coherent mental model of their content. According to the construction-integration model of text comprehension (10, 22), text processing comprises three levels of analysis: the surface structure, the text base with its propositional structure, and the mental model. The surface structure represents the exact wording of the text and its syntactics. The text base represents the propositional structure of the text. Based on this propositional text base structure, readers search for relations and, if not directly mentioned in the text, make bridging inferences and knowledge-based inferences in order to establish a coherent mental model. The more coherent a text base is, that is, the more its arguments overlap and related elements are directly linked in the text, the fewer inferences are needed to establish a coherent mental model of its content. Furthermore, the coherence of a text is much more important for readers and listeners with low prior knowledge and therefore with less ability to generate inferences than for readers and listeners with high prior knowledge (23).

Regarding text coherence, a distinction must be made between local and global coherence (22). Local coherence is established if currently processed information is automatically connected with the immediately preceding context still present in working memory. Global coherence, on the other hand, involves relations between currently processed information and information presented earlier in the text which is no longer present in working memory (24). In this case, readers and listeners have to search their episodic text memory for possible related antecedents to the currently processed information and reinstate it in working memory in order to relate both to each other and thereby understand the text. Establishing global coherence is cognitively more effortful than establishing local coherence. Accordingly, it could be shown empirically that readers detected inconsistencies primarily when presented locally but not globally coherent. This indicates an easier co-activation and integration of information that is presented temporally close than information presented temporally distant (25). Hence, texts presenting related information temporally close to each other (local coherence) should be better understandable than texts presenting related information temporally distant to each other (global coherence).

The present study aims to bring together both lines of research, that is, the visual processing of more or less spatially distant, but conceptually related elements in paintings, and the verbal processing of these elements if they are named in a concurrent audio text in a more or less temporally distant manner.

More specifically, against the background of the findings on the visual world paradigm (26), it is assumed that viewers tend to follow the sequence of a verbal description during inspection of a picture. Accordingly, such a description can be used to perceptually and cognitively link semantically related but spatially separated picture elements. Glaser and Schwan (2), for example, could show that by sequentially locating, naming, and explaining picture elements in an audio text, viewers tend to fixate these elements so that the verbal explanation as a whole induces an inter-individually homogenous viewing behavior and directs the gaze of the observers along a particular sequence across the picture elements and thereby influences the order in which picture elements are processed and stored in memory.

The close connection of the perceived picture scene, its verbal description, and its visual mental representation is also confirmed by research on visual mental imagery (e.g. 27, 28) as well as by studies in the context of the blank screen paradigm. In the latter, a picture is verbally described, and the subjects are requested to imagine the picture or are either shown pictures and then are requested to describe them from memory. In both cases, their eye movements are recorded while looking at an empty white area (6, 7). It has been shown that the fixations and saccades on the white surface reflect the structure of the scene or the sequence of their verbal description. In addition, there was a positive correlation between the precision of blank screen fixation and the memory performance of the picture elements (29).

Higher text coherence may therefore not only foster the generation of cognitive linkages between different textual elements and the generation of a coherent mental model of the text but may also enhance perceptual linkage of visual elements by reducing cognitively effortful visual search processes. By naming picture elements and their particular relation temporally close (local coherence) instead of temporally distant (global coherence) to each other, related picture elements should be found faster and therefore fixated more often. Accordingly, an enhanced perceptual linkage should be manifest by a higher number of backward fixations from the second to the first of the named picture elements similar to lookback fixation time in the text processing research by Hyönä, Lorch, and Rinck (30).

### Hypotheses

Based on the above described literature, we assume that, on a perceptual level, the number of backward fixation counts should be higher for spatially close than for spatially distant picture elements (H1a), and also in the case of naming these related picture elements, the number of backward fixation counts should be higher for temporally close than temporally distant elements (H1b).

On a semantic deep processing level, we assume that the retention of related picture elements and their relations in a free recall task should be higher for spatially close than for spatially distant picture elements (H2a), and also in the case of naming these related picture elements, it should be higher for temporally close than temporally distant elements (H2b).

## Methods

### Participants

In the present study, 56 participants were examined. Thereof, twelve participants were excluded because they had heard the wrong audio text version (programming mistake). Further, four participants were excluded because their eye recording deviation was greater than 0.80 degrees; two participants were excluded because they answered the free recall test for the wrong painting; one participant was excluded because of knowing at least one of the two test paintings, and another participant was excluded because of not understanding German on a native speaker level. From the remaining 36 participants analyzed, 29 were female, four were male, and three were diverse. The participants were between 18 and 27 years old (*M* = 21.89, *SD* = 2.82).

### Design

The hypotheses were tested in a 2x2x2 design with spatial distance (distant vs. close), temporal distance (distant vs. close), and painting (Leutze vs. West) as within-subject variables. Analyses of variance (ANOVA) were calculated to test our hypotheses.

### Materials

A 250 Hz remote eye-tracking system and the eye-tracking software IView RED 4.4 from the company Senso Motoric Instruments (SMI) were used. The eye tracking camera was mounted below 23-inch Dell monitors (1920x1080px) of two computers on which the presentation was done with SMI Experiment Center 3.7.68. The participants were seated about 68 cm in front of the screen and their chins were placed on a chin rest to control for constant eye-to-screen distance and head movements. They navigated with the keyboard through the experiment. Audio explanations were presented via a headset. The eye tracking data analysis was performed with SMI BeGaze 3.7.59.

**Figure 1.**
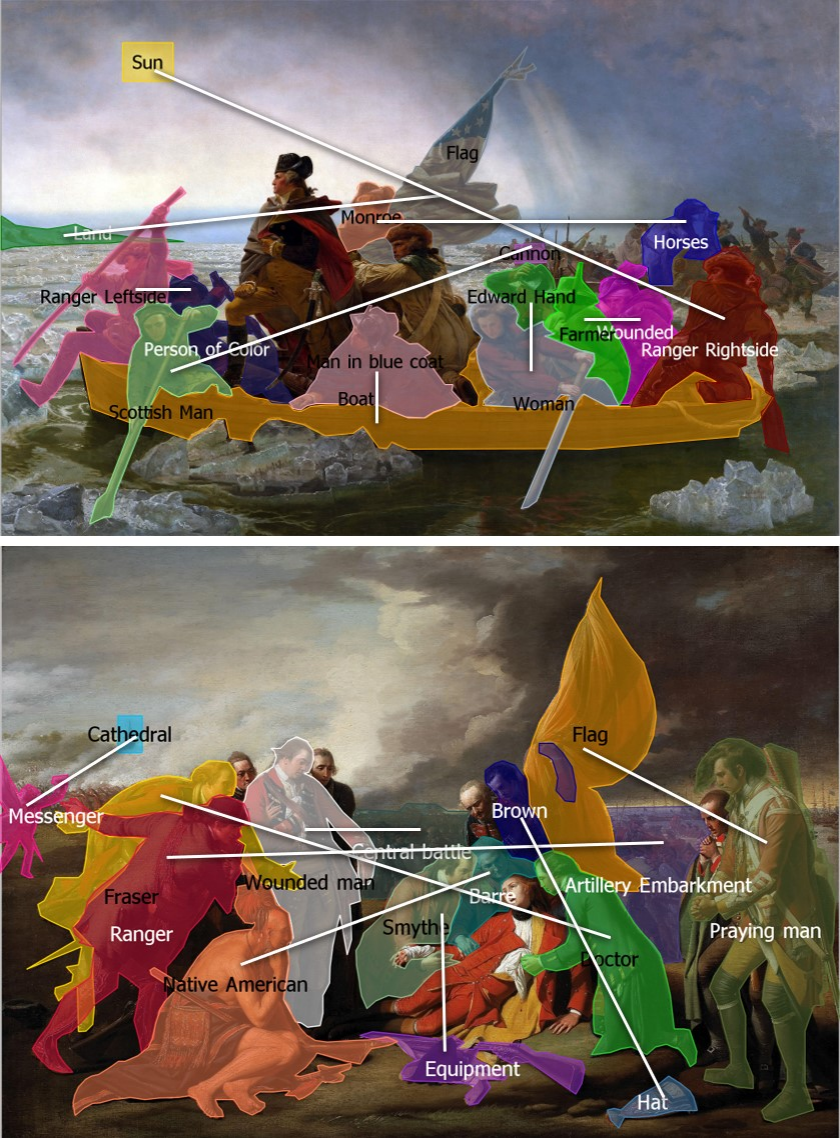
Spatially close and distant picture elements of the Leutze painting (above) and spatially close and distant picture elements of the West painting (below). Source of the Leutze painting: https://upload.wikimedia.org/wikipedia/commons/9/95/Washington_Crossing_the_Delaware_by_Emanuel_Leutze%2C_MMA-NYC%2C_1851.jpg, licensing: public domain. Source of the West painting: https://upload.wikimedia.org/wikipedia/commons/4/4f/Benjamin_West_005.jpg, licensing: public domain.

Four history paintings were presented on the computer screen, with two of them used as flanking paintings (“The Death of Socrates” by Jacques Louis David and “The Proclamation of the German Emperor” by Anton von Werner) and two as testing material (“Washington Crossing the Delaware” by Emanuel Leutze and “The Death of General Wolfe” by Benjamin West). From each of the test paintings, four picture element pairs that were spatially close and four picture element pairs that were spatially distant to each other were chosen as test items (see also Figure 1). The definition of these picture elements was made as follows: The paintings were a priori divided into single meaningful entities such as persons, objects (like a boat or a banner), or landscape details (like the shore or a distant cathedral) which, according to the art historical literature, are of importance for understanding the paintings and the artists’ intentions. The main character of each painting was excluded because they were already named in the title of the pictures. From the Leutze painting, the signature was also excluded due to being extra-diegetic. From the Wests painting, very small and not clearly identifiable picture elements (e.g. a man falling from a horse in the background of the painting) were also excluded. After this procedure, 20 picture elements from the Leutze and 16 picture elements from the West painting remained. Saliency of these picture elements was measured in a prior study (*n* = 12) in which participants watched the paintings in a free viewing mode for 30 seconds without any other information. Saliency was operationalized by the mean fixation time in milliseconds on each of the picture elements divided by the size of the respective picture element in pixel multiplied by factor 100 for better readability. The 20 picture elements from the Leutze painting and the 16 picture elements from the West painting were then classified in high-saliency and low-saliency picture elements by applying a median split on their saliency values. From the above identified picture elements of each painting, 4 spatially close (2 pairs with high-saliency and 2 with low-saliency picture elements) and 4 spatially distant (2 pairs with high-saliency and 2 with low-saliency picture elements) picture element pairs were defined.

For each of the flanking paintings, one audio text was created, and for each of the test paintings, two versions were created. The two versions had the same content naming and describing the eight picture element pairs (test items) and their relations but differed due to the temporal distance of their presentation in the audio text: In Version A, two spatially close pairs and two spatially distant pairs were presented temporally close and two other spatially close and two spatially distant pairs were presented temporally distant. In Version B, the pairs that were presented temporally close in Version A were now presented temporally distant and the pairs presented temporally distant in Version A were presented temporally close. In both versions, the elements of a picture element pair were always presented in the same order, except for two spatially distant pairs from the West painting. Here, due to a technical mistake the pairs in Version B were presented in reverse order of Version A. The naming of each picture element had a three-part structure of localization (where is it located in the painting), description (what does it look like) and interpretation (how is it related to the historical event). In the last sentence of the interpretation, information that related the respective picture element to its partner element was given.

An example of a temporally close presentation of picture elements in the audio texts of the present study is as follows: “In the center of the picture behind Wolfe [localization] is a man with black hair, looking at his dying general [description]. It is Henry Browne, a lieutenant in the Grenadier Regiment which was highly regarded with its elite soldiers [interpretation]. Grenadiers, like Henry Brown, usually wore special headgear as a distinguishing mark [relation]. At the bottom right of the picture [localization] is a hat richly decorated [description]. It is a so-called miter [interpretation]. Such a miter was usually worn by grenadiers as a distinguishing mark [relation].”

Temporally distant presentations of the related picture elements were formulated in the same way but with other picture elements explained in between: “In the center of the picture behind Wolfe [localization] is a man with black hair, looking at his dying general [description]. It is Henry Browne, a lieutenant in the Grenadier Regiment which was highly regarded with its elite soldiers [interpretation]. Grenadiers, like Henry Brown, usually wore special headgear as a distinguishing mark [relation]. […] At the bottom right of the picture [localization] is a hat richly decorated [description]. It is a so-called miter [interpretation]. Such a miter was usually worn by grenadiers as a distinguishing mark [relation].”

The audio text versions were equally long: 7:12 minutes for the Leutze painting and 7:14 minutes for the West painting. The temporal distance was kept constant by the amount of picture elements explained in between: Either the related picture elements were explained in succession (temporally close) or with seven picture elements explained in between (temporally distant).

### Measures

Eye tracking data was collected during the presentation of the paintings. From this data, we analyzed the backward fixation counts on the first of the two mentioned related picture elements of each painting occurring after the second elements and their relations to the first elements were mentioned in the audio explanation. In order to do that, for each of these picture elements mentioned first, an Area Of Interest (AOI) was defined, and fixation counts on these AOIs were measured from the beginning of the relating sentence of the second mentioned picture element, continuing until the explanation of the next picture element started.

In a free recall test, the participants were asked to write down those related pairs and their relations that they remembered for the Leutze and the West painting. The time for this task was limited to eight minutes for each painting. The participants could achieve one point for each correctly recalled pair and another point for recalling their correct relation. Sum scores were calculated; the learners could thus achieve 0-16 points in the recall test of each of the two test paintings.

### Procedure

One to two participants were tested in each session. First the participants were welcomed and seated in front of the computer screen. The eye-tracking device was adjusted based on a 9-point calibration. After a short instruction about the experiment, the four paintings were presented sequentially together with their respective audio explanations, each introduced by a written introduction stating in German: “In the following, you can see the painting [title of the painting] by the artist [name of artist]”. The first painting was “The Death of Socrates”, followed in a counterbalanced way by either “Washington Crossing the Delaware” or “The Death of General Wolfe”. The last painting was “The Proclamation of the German Emperor”. After the audio-visual presentation, a filler task of about 10 minutes followed in order to inhibit further memorization of the previously seen information. This filler task was a memory puzzle in which word-picture-pairs had to be build. Then, the free recall test was handed out to the participants in a balanced way, either about the Leutze or the West painting. Finally, demographics (age, gender, and profession) were collected. The participants were asked whether they were familiar with any of the paintings that were presented previously and if yes which one. They were then debriefed and paid 10 Euros for their participation that took about 70 minutes.

## Results

### Eye movements

Firstly, in order to check whether the naming of the picture elements in the audio explanation did direct the viewers’ visual attention to the respective elements, as shown by Glaser and Schwan (2), for each picture element of each painting a t-test was calculated, comparing the fixation time (in ms) on its AOI during the time of naming it versus during a predefined time of non-naming. The end of the description part of each picture element was defined as the beginning of the time of naming because from this point of time on, the respective picture element can be clearly identified and willingly attended to in the painting. From this time point on, the following 7 seconds, in which the respective picture element was interpreted by the audio text, were used as the time period of analysis. For the times of non-naming, a 7 second time period during an introduction sentence about the physical qualities (materials, height and width) of the painting was defined as the time period of analysis. The 16 t-tests were Bonferroni corrected for each painting. Results showed that all t-test were significant for the Leutze (all p < .001) and for the West painting (all p < .001). For each comparison, fixation times on the AOIs of picture elements at times of naming were significantly longer than at times of non-naming. Hence, the information in the audio texts seems to have been sufficient to clearly identify and visually localize the named picture elements. The attention guiding effect of naming pictorial elements was thus confirmed.

With regard to our hypotheses, an analysis of variance (ANOVA) was calculated with spatial distance of elements in the painting (distant vs. close), temporal distance of their naming in the audio text (distant vs. close), and painting (Leutze vs. West) as within-subjects variables. The ANOVA revealed a significant main effect of spatial distance, F(1, 35) = 27.52, p < .001, η_p_² = .440. The pairs that were spatially close (M = 2.25, SD = 1.45) showed more backward fixation counts than pairs that were spatially distant (M = 0.93, SD = 1.08). There was no main effect of temporal distance of naming in the audio text on backward fixation counts (close: M = 1.51, SD = 1.27; distant: M = 1.67, SD = 1.08), F(1, 35) = 0.67, p = .419, η_p_² = .019, and also no main effect of painting (West: M = 1.56, SD = 1.52; Leutze: M = 1.63, SD = 1.04), F(1, 35) = 0.07, p = .794, η_p_² = .002. There was a significant two-way interaction of spatial distance and painting, F(1, 35) = 23.47, p < .001, η_p_² = .401. For the Leutze painting, the spatially close pairs (M = 2.76, SD = 1.83) showed a significantly higher amount of backward fixations counts than the spatially distant pairs (M = 0.49, SD = 0.94), p < .001, whereas for the West painting, the spatially distant pairs (M = 1.38, SD = 1.56) did not significantly differ from the spatially close pairs (M = 1.73, SD = 1.95) with regard to backward fixation counts, p = .231. There was neither a significant two-way interaction of spatial distance and temporal distance, F(1, 35) = 0.16, p = .689, η_p_² = .005, nor a significant two-way interaction of temporal distance and painting, F(1, 35) = 0.25, p = .624, η_p_² = .007. There was also no significant three-way interaction of spatial distance, temporal distance and painting, F(1, 35) = 1.23, p = .275, η_p_² = .034. Mean values and standard deviations for this analysis can be seen in Table 1. The results partially confirm Hypothesis H1a that the number of backward fixation counts should be higher for spatially close than for spatially distant picture ele-ments, but not H1b that backward fixation counts should be higher in the case of naming the related picture elements temporally close rather than temporally distant to each other.

**Table 1:** Mean values and standard deviations (in parentheses) of backward fixation counts in the ANOVA with spatial distance (distant vs. close), temporal distance (distant vs. close), and painting (Leutze vs. West) as within-subjects variables.

	Leutze		West	
	spatially close	spatially distant	spatially close	spatially distant
temporally close	2.53 (2.69)	0.44 (1.32)	1.94 (3.39)	1.14 (1.79)
temporally distant	3.00 (2.53)	0.53 (1.11)	1.53 (1.72)	1.61 (2.59)

### Free recall

An analysis of variance (ANOVA) was calculated with spatial distance of elements in the painting (distant vs. close), temporal distance of their naming in the audio text (distant vs. close), and painting (Leutze vs. West) as within-subjects variables. The ANOVA revealed neither a significant main effect of spatial distance, F(1, 35) < 0.01, p = .959, η_p_² < .001, nor of temporal distance, F(1, 35) = 0.75, p = .394, η_p_² = .021. The main effect of painting was significant, F(1, 35) = 9.83, p = .003, η_p_² = .219. The related pairs and their relations of the Leutze painting (M = 1.32, SD = 0.85) were retained better than the related pairs and their relations of the West painting (M = 0.88, SD = 0.72). There was no significant interaction for spatial distance and temporal distance, F(1, 35) = 1.27, p = .267, η_p_² = .035. The interaction for temporal distance and painting was also not significant, F(1, 35) = 0.33, p = .569, η _p_² = .009. The interaction of spatial distance and painting was significant, F(1, 35) = 22.09, p < .001, η_p_² = .387. For the Leutze painting, the related pairs and their relations of spatially close pairs (M = 1.61, SD = 1.12) were retained better than the related pairs and their relations of the spatially distant pairs (M = 1.04, SD = 1.02), p = .012, but for the West painting, the related pairs and their relations of spatially distant pairs (M = 1.16, SD = 0.85) were retained better than those of the spatially close pairs (M = 0.61, SD = 0.82), p < .001. The three way interaction of spatial distance, temporal distance, and painting was not significant, F(1, 35) < 0.01, p = .955, η_p_² < .001 Mean values and standard deviations for this analysis can be seen in Table 2. The results, therefore, partially confirm Hypothesis H2a that the retention of related picture elements in a free recall task should be higher for spatially close than for spatially distant picture elements, but not H2b that retention should be better in the case of naming related picture elements temporally close than temporally distant to each other.

Furthermore, the number of backward fixations counts and retention test scores across all first named elements of picture element pairs and across all paintings were positively correlated and showed a small, but significant Spearman-Rho correlation of r_s_ = .212, p < .001.

**Table 2:** Mean values and standard deviations (in parentheses) of retention of related pairs and their relations in the ANOVA with spatial distance (distant vs. close), temporal distance (distant vs. close), and painting (Leutze vs. West) as within-subjects variables.

	Leutze		West	
	spatially close	spatially distant	spatially close	spatially distant
temporally close	1.50 (1.48)	1.08 (1.38)	0.42 (0.94)	1.14 (1.22)
temporally distant	1.72 (1.34)	1.00 (1.22)	0.81 (1.26)	1.19 (1.35)

## Discussion

### Summary of the results

The present study examined the influence of the spatial distance between conceptually related picture elements and the textual coherence of their naming in a concurrent verbal description on visual processing and memory. In two paintings, pairs of picture elements were either spatially close or distant to each other and described verbally either temporally close or distant in an accompanying audio text. Regarding viewing behaviour, it was hypothesized that the number of backward fixation counts should be higher for spatially close than for spatially distant picture elements (H1a).

The results showed a higher amount of backward fixation counts for spatially close compared to spatially distant pairs, but only for the Leutze painting. Hence, Hypothesis H1a was only partially confirmed. One possible reason for the missing effect of spatial distance for the West painting may be that the spatial distances between the distant picture elements in the West painting were smaller than in the Leutze painting, and the spatial distances between the close picture elements were larger in the West than in the Leutze painting (see also Figure 1). Hence, the manipulation of spatially close and distant picture elements was weaker in the West than in the Leutze painting. Accordingly, mean values lay in the expected direction but did not reach significant differences.

It was also hypothesized that backward fixation counts should be higher in the case of naming the related picture elements temporally close rather than temporally distant to each other (H1b).

The analysis comparing times of naming and not naming picture elements in the audio explanation with regard to fixation times on these picture elements showed that named picture elements were actually fixated immediately after their naming. Hence, information in the audio texts seems to have been sufficient to clearly identify and visually localize the named picture elements and guide the viewers’ attention. However, there was no effect of temporal distance on the perceptual linkage of the related picture elements, and therefore, Hypotheses H1b was not confirmed by the present study. While naming did guide the viewers visual attention to particular picture elements (2), audio text coherence did not lead to corresponding eye movements perceptually connecting the related picture elements in the form of backward fixations. One possible reason for this lack of visual interconnection could be that the viewers in the present study failed to establish textual coherence between the named picture element pairs in first place. While the contingency of backward fixations on the establishment of textual coherence is demonstrated by the positive correlation between the two measurements, the low number of remembered picture pairs and the lack of effects of the temporal distance of naming related elements indicate that, in many cases, the establishing of textual coherence did not take place.

Regarding memory, it was hypothesized that the retention of related picture elements in a free recall task should be higher for spatially close than for spatially distant picture elements (H2a).

In general, the number of recalled pairs was rather low. On average less than one half of the pairs were recalled. Also, the retention of related pairs and their relations for the Leutze painting was better than retention of the related pairs and their relations for the West painting in the present study. In line with Hypothesis H2a, spatially close pairs of picture elements were retained better than spatially distant pairs for the Leutze painting. However, for the West painting, the opposite result was found, namely, that spatially distant pairs of picture elements were retained better than spatially close pairs. Hence, Hypothesis H2a could only be partially confirmed. An explanation for the opposite effect of spatial distance with regard to the West painting cannot be due to the audio explanation since there was no main effect or interaction with temporal distance which could have directed visual attention and influenced memory differently for spatially close and distant picture element pairs. Also, the audio information seems to have been precise enough to clearly identify and visually localize the named picture elements since the naming effect occurred for each AOI. Hence, confusion about identifying certain picture elements did not occur and can therefore not be regarded as an explanation for the opposite results for the two paintings on the free recall of spatially close and distant elements. Furthermore, if we assume perceptual linkage to be a prerequisite for cognitive linkage, then in the case of the non-significant effects of spatial distance on the number of backward fixation counts for the West painting, we would have expected a non-significant influence of spatial distance on the recall of the picture element pairs and their relations in the West painting and not an opposite effect as it occurs in the present study. Additionally, if perceptual linkage as a prerequisite failed to direct processing, maybe visual characteristics could unfold their influence, leading to a better retention of spatially distant compared to spatially close picture elements and their relations for the West painting. Therefore, the opposite effect of spatial distance on retention for the West painting may instead be explained by visual characteristics of the picture element pairs. One such visual characteristic is the saliency of the picture elements. In line with the saliency map hypothesis, postulating that fixations should be attracted to picture elements of higher saliency (31), we tried to control this aspect in our study by dividing the picture elements into picture elements of low and high saliency, using a median split in a prior study. However, this is only one of many potential picture characteristics and maybe other methods such as mathematical models simulating bottom-up processing or brain imaging techniques in combination with eye tracking, as discussed by Rayner ( 32), may have been more appropriate to control for processing the paintings based on picture characteristics. Furthermore, besides this visual aspect of the paintings, it could be possible that the picture elements of the two paintings may have differed with regard to their meaning to the participants and that this, according to Henderson and Hayes (33), may have played the dominant role in guiding the viewers’ processing of the paintings and caused the different results for the two paintings.

It was also assumed that memory should be better in the case of naming related picture elements temporally close than temporally distant to each other (H2b). There were no effects of the temporal distance of verbal descriptions on the retention of related pairs of picture elements. Consequently, Hypothesis H2b could not be confirmed by the present study. It seems that viewers were not able to sufficiently establish relations on a textual level, neither for temporally close nor for temporally distant picture element pairs. This is contrary to prior research (22, 24), but may be explained by the use of transient audio explanations in the present study instead of written text material often used in prior research. Processing picture elements and their relations in an audio explanation may have been too effortful to result in cognitive linkage and corresponding eye movement behaviour. Maybe the results on memory could have been found with permanent written explanations instead of transient audio ones. However, in this case, perceptual linkage could not have been examined because while reading the text, perceptual linkage of the elements in the picture would not be possible. Thus, studies with audio-visual material of lesser complexity are needed to examine this issue.

A further reason why we could not find effects of text coherence on perceptual and cognitive linkage between related picture elements may be that in our study situation the painting and not the audio text was the main learning focus. Therefore, participants may have had difficulty recognizing and memorizing the relations between pairs of picture elements presented in the audio text because their mental capacities were more focused on the painting than on the audio explanation.

### Limitations

While using historical paintings allowed for a high ecological validity of the pictorial material, the downside consequence was that the spatial distance of the picture elements could not be varied systematically but was predefined by the paintings; that is, the same picture element pairs could not be presented either spatially distant or spatially close. Hence, the composition of the paintings may have had an influence on the present results. We tried to control for this by analysing two instead of only one painting. Due to practical reasons, we did not use a greater number of paintings in our study, although we were aware that this may limit the generalizability of our findings. And actually, with regard to eye movements and retention, our effects did indeed depend on the examined painting. The present results should therefore be replicated with a larger sample of diverse paintings.

Additionally, the present study contained only one experiment. Further studies are therefore needed to replicate the present findings in other picture viewing situations with other historical paintings and with other pictorial material such as complex maps (e.g. architectural plans or plans of electric circuits that professionals have to deal with in their everyday work) or pictures of real-life scenes (photographic evidence within court hearings or satellite pictures for tactical, military decisions). Such studies are needed in order to examine whether the cognitive processes found in the present study are also valid beyond processing artworks in a laboratory setting. The present study was conducted in a laboratory with relatively long audio texts, similar to conditions found in TV art documentaries (34). In contrast, museums and art exhibitions typically operate with much shorter audio guide texts. Therefore, further research should examine the present research questions in the field of art galleries. Here, the influence of the museum context including additional information about the contents of the paintings can be considered and the audio texts shortened. Furthermore, the present study can only draw conclusions for a restricted population relatively young in age, with an academic background and low prior knowledge. We did not measure individual differences and therefore cannot arrive at any conclusions on how the examined effects influence types of learners differently, for example, dependent on prior knowledge, learning abilities, amount of attentional resources, working memory capacity, motivation, or interest. Additionally, we did not consider the contextual and emotional factors in the information-processing stage model of aesthetic processing by Leder et al. (8) in the present study.

Nevertheless, the result indicate that the spatial distance of picture elements matters with regard to the perceptual linkage of information. With the results for one of the two paintings, the present study confirms prior research about the spatial contiguity effect on perceptual (14, 15) and cognitive linkage (4, 11, 12, 13), and doing so, used the measure of backward fixation counts, which to our knowledge is used rather seldom (e.g. 30) Furthermore, the present study expands prior research about the spatial contiguity effect from multimedia research often examining text-picture relations in the field of natural science to processing related pairs of picture elements in the field of art reception (8).

Since we could not find the expected influence of temporal distance on perceptual and cognitive linkage, unfortunately, prior research on text coherence (5, 10, 22, 23, 24) could not be expanded to a picture processing situation in which the painting was the main learning focus and the transient audio text functioned as an explanation of the painting. While we could not find an influence of audio text coherence on visual attention and retention of relations in paintings, our results nevertheless demonstrate that audio explanations strongly guide the viewers’ visual attention to the named picture elements. This is important for practitioners such as audio guide designers and museum curators. Audio explanations should be designed in a way that all picture elements that are intended to be attended to are actually mentioned. Mentioning these picture elements in an audio explanation should be done clearly. Similarly, personal guides should distinctly mention the picture elements that should be attended to by the visitors. Furthermore, multimedia guides can improve this attention guiding process by additional signals functioning as visual cues. Such visual cues may be frames or zoom-ins that highlight particular picture elements and thereby foster verbal cueing (34).

## Ethics and Conflict of Interest

The authors declare that the contents of the article are in agreement with the ethics described in http://biblio.unibe.ch/portale/elibrary/BOP/jemr/ethics.html and that there is no conflict of interest regarding the publication of this paper.

## Acknowledgements

This research was supported by grant 638221 from the German Research Foundation.
